# Pure Wisdom or Potemkin Villages? A Comparison of ChatGPT 3.5 and ChatGPT 4 on USMLE Step 3 Style Questions: Quantitative Analysis

**DOI:** 10.2196/51148

**Published:** 2024-01-05

**Authors:** Leonard Knoedler, Michael Alfertshofer, Samuel Knoedler, Cosima C Hoch, Paul F Funk, Sebastian Cotofana, Bhagvat Maheta, Konstantin Frank, Vanessa Brébant, Lukas Prantl, Philipp Lamby

**Affiliations:** 1 Department of Plastic, Hand and Reconstructive Surgery University Hospital Regensburg Regensburg Germany; 2 Division of Hand, Plastic and Aesthetic Surgery Ludwig-Maximilians University Munich Munich Germany; 3 Division of Plastic Surgery Brigham and Women's Hospital Harvard Medical School Boston, MA United States; 4 Department of Otolaryngology, Head and Neck Surgery School of Medicine Technical University of Munich Munich Germany; 5 Department of Otolaryngology, Head and Neck Surgery University Hospital Jena Friedrich Schiller University Jena Jena Germany; 6 Department of Dermatology Erasmus Hospital Rotterdam Netherlands; 7 Centre for Cutaneous Research Blizard Institute Queen Mary University of London London United Kingdom; 8 College of Medicine California Northstate University Elk Grove, CA United States; 9 Ocean Clinic Marbella Spain

**Keywords:** ChatGPT, United States Medical Licensing Examination, artificial intelligence, USMLE, USMLE Step 1, OpenAI, medical education, clinical decision-making

## Abstract

**Background:**

The United States Medical Licensing Examination (USMLE) has been critical in medical education since 1992, testing various aspects of a medical student’s knowledge and skills through different steps, based on their training level. Artificial intelligence (AI) tools, including chatbots like ChatGPT, are emerging technologies with potential applications in medicine. However, comprehensive studies analyzing ChatGPT’s performance on USMLE Step 3 in large-scale scenarios and comparing different versions of ChatGPT are limited.

**Objective:**

This paper aimed to analyze ChatGPT’s performance on USMLE Step 3 practice test questions to better elucidate the strengths and weaknesses of AI use in medical education and deduce evidence-based strategies to counteract AI cheating.

**Methods:**

A total of 2069 USMLE Step 3 practice questions were extracted from the AMBOSS study platform. After including 229 image-based questions, a total of 1840 text-based questions were further categorized and entered into ChatGPT 3.5, while a subset of 229 questions were entered into ChatGPT 4. Responses were recorded, and the accuracy of ChatGPT answers as well as its performance in different test question categories and for different difficulty levels were compared between both versions.

**Results:**

Overall, ChatGPT 4 demonstrated a statistically significant superior performance compared to ChatGPT 3.5, achieving an accuracy of 84.7% (194/229) and 56.9% (1047/1840), respectively. A noteworthy correlation was observed between the length of test questions and the performance of ChatGPT 3.5 (ρ=–0.069; *P*=.003), which was absent in ChatGPT 4 (*P*=.87). Additionally, the difficulty of test questions, as categorized by AMBOSS hammer ratings, showed a statistically significant correlation with performance for both ChatGPT versions, with ρ=–0.289 for ChatGPT 3.5 and ρ=–0.344 for ChatGPT 4. ChatGPT 4 surpassed ChatGPT 3.5 in all levels of test question difficulty, except for the 2 highest difficulty tiers (4 and 5 hammers), where statistical significance was not reached.

**Conclusions:**

In this study, ChatGPT 4 demonstrated remarkable proficiency in taking the USMLE Step 3, with an accuracy rate of 84.7% (194/229), outshining ChatGPT 3.5 with an accuracy rate of 56.9% (1047/1840). Although ChatGPT 4 performed exceptionally, it encountered difficulties in questions requiring the application of theoretical concepts, particularly in cardiology and neurology. These insights are pivotal for the development of examination strategies that are resilient to AI and underline the promising role of AI in the realm of medical education and diagnostics.

## Introduction

Since its inception in 1992, the United States Medical Licensing Examination (USMLE) has been considered an integral milestone in medical education [1]. The 3 USMLE steps are jointly sponsored by the Federation of State Medical Boards and the National Board of Medical Examiners. Each step is designed to specifically test another facet of the examinee’s skill set. For instance, USMLE Step 1 assesses a student’s understanding and application of basic sciences relevant to the field of medicine (eg, anatomy and physiology), while USMLE Step 2 tests the examinee’s clinical knowledge (USMLE Step 2 CK) and communication skills (USMLE Step 2 CS). USMLE Step 3 evaluates the student’s understanding of biomedical and clinical science [2-4]. USMLE scores have been associated with residency matching and future career perspectives [5].

Artificial intelligence (AI)–supported tools have been proposed for a variety of medical scenarios, including preoperative outcome simulation, patient education, and automated disease grading [6-9]. Recently, chatbots such as ChatGPT have emerged as next-generation AI technology. The strengths of this novel AI-powered approach include 24-7 availability, cost efficiency, and individualization [10]. A mounting body of evidence has investigated ChatGPT’s performance on different standardized exams. For instance, Hoch et al [11] reported that ChatGPT answered 57% of facial surgery board certification test questions correctly, while Kung et al [12] used a limited set of USMLE test questions (USMLE Step 1: 119; USMLE Step 2 CK: 102; USMLE Step 3: 122) and found that ChatGPT achieved performance levels near the passing threshold for all 3 steps.

However, there is a scarcity of studies that comprehensively investigate overall ChatGPT performance on USMLE Step 3 test questions in a large-scale study and compare test performances between ChatGPT 3.5 and ChatGPT 4. This knowledge gap may increase the risk of AI cheating in such career-deciding exams and cloud the vision of ChatGPT’s strengths and limitations.

Therefore, we aimed to determine ChatGPT’s performance on USMLE Step 3 practice test questions based on 1840 AMBOSS USMLE Step 3 Style Questions. This line of research may serve as a primer elucidating the strengths and weaknesses of multiple ChatGPT versions and deducing evidence-based strategies to counteract AI cheating.

## Methods

### Access to Question Bank and Data Entry Procedure

From June 12, 2023, to June 19, 2023, we obtained access to the AMBOSS question bank [13]. Within this time frame, we collected a total of 1840 practice questions specifically designed for the USMLE Step 3 exam. Before initiating our study, we acquired official permission from AMBOSS (AMBOSS GmbH) to use their USMLE Step 3 question bank for research purposes. To ensure the reliability of our data, 2 examiners (MA and LK) cross-checked the question inputs randomly to confirm that none of the answers were indexed on Google before June 19, 2023. Many USMLE questions are on the internet, including USMLE sample questions as well as a few AMBOSS questions; however, we ensured that those questions were not included in this analysis to minimize the risk of prior memorization of the questions by ChatGPT. July 19, 2023, was chosen since it represents the most recent accessible date within the training data set of ChatGPT. There are many forms of AI versions with capabilities to answer USMLE Step 3 practice test questions; however, ChatGPT is the most widely used AI at the time of this study, making it the best fit for our study.

### Question Screening and Categorization

To maintain the quality of our sample questions, we subjected all test questions to independent screening by 4 examiners (MA, SK, CCH, and LK). Questions containing clinical images and photographs were excluded from the study, resulting in the removal of 229 image-based questions. Subsequently, the remaining 1840 test questions were classified based on their respective specialties, using the categorization provided by AMBOSS. All questions included in our study followed a multiple-choice single-answer format. The questions used for both ChatGPT 3.5 and ChatGPT 4 were matched for content and difficulty based on the standardized definitions provided by the AMBOSS question bank to ensure consistent analysis between both AI versions.

### Comparison of ChatGPT Versions and Analysis of Question Stems

To evaluate any performance differences between ChatGPT 3.5 and ChatGPT 4, we conducted a subgroup analysis specifically focusing on ChatGPT 4. Additionally, we analyzed the question stems of both ChatGPT 3.5 and ChatGPT 4, specifically looking for specific buzzwords related to diagnostic methods and patient information, such as “Ultrasound,” “Serology,” and “Nicotine Abuse.” These particular words and phrases may suggest one answer over another and thus are essential for test-taking. For example, if the question states “Nicotine Abuse,” which is suggestive of cigarette or tobacco use, the patient in the question stem is more likely to have cancer. The purpose of this analysis was to identify any variations in accuracy based on the presence of these factors. Furthermore, we assessed performance differences between ChatGPT 3.5 and ChatGPT 4 based on the length of the test questions.

### Assessment of Question Difficulty

To assess the difficulty of the test questions, we used the proprietary rating system of the AMBOSS question bank. This system assigns a difficulty level to each question based on a scale of 1 to 5 hammers. A rating of 1 hammer corresponds to the easiest 20% of questions, while 5 hammers indicate the most challenging 5% of questions.

### Data Entry Process

One examiner (MA) manually inputted the test questions into ChatGPT. The questions were transcribed verbatim from the AMBOSS question bank, preserving the original text and answer choices. To ensure the integrity of ChatGPT’s performance, no additional prompts were introduced intentionally by the authors, thereby minimizing the potential for systematic errors. Each question was treated as a separate chat session in ChatGPT to minimize the impact of memory retention bias. As an example, the following provides a standard test question from the category “Competency: Patient Care Content Area: General Principles”:

What is the most suitable course of action to take next in the case of a 54-year-old man, previously in good health, who presents to the emergency department after being bitten by a stray dog in South America? The bite punctured his right leg, but he has diligently cleaned the wound daily with soap and peroxide. The patient is not experiencing pain, fever, or chills, and his vital signs are normal. The examination reveals healing puncture wounds with minimal redness, and there is no fluctuation or palpable lymph nodes in the groin. The patient had a tetanus booster vaccination three years ago.

(A) Provide rabies vaccination

(B) Administer tetanus immune globulin

(C) Request cerebrospinal fluid analysis

(D) Order an MRI [magnetic resonance imaging] scan of the brain and spinal cord

(E) No immediate action is required at this time

### Recording and Evaluation of ChatGPT Responses

The answers generated by ChatGPT were documented and incorporated into the corresponding AMBOSS USMLE Step 3 practice question. Subsequently, we systematically gathered and recorded information regarding the accuracy of these responses in a separate data spreadsheet.

### Statistical Analysis

We used the Pearson chi-square test to determine differences in question style and categories. Bivariate correlation analysis between ChatGPT performance, test question length, and difficulty was conducted using the Spearman correlation coefficient (ρ). IBM SPSS Statistics 25 (IBM Corp) was used for statistical analysis, and a 2-tailed *P* value ≤.05 was considered statistically significant.

## Results

### General Test Question Characteristics and Performance Statistics

The overall accuracy of ChatGPT 3.5 for USMLE Step 3 was 56.9% (1047/1840), while ChatGPT 4 answered 84.7% (194/229) of test questions correctly (*P*<.001). Specialty-specific number of test questions and performance scores are presented in Tables 1 and 2. ChatGPT 3.5 received the greatest number of questions on the nervous, cardiovascular, and gastrointestinal systems, while ChatGPT 4 received the greatest number of questions on behavior health, the female reproductive system, as well the blood and lymphatic system. When considering the accuracy of ChatGPT based on the category of questions, ChatGPT 3.5 performed the best on behavioral health, multisystem processes and disorders, and pregnancy-related questions. On the other hand, ChatGPT 4 had the greatest accuracy on questions related to the endocrine and musculoskeletal systems as well as biostatistics and multisystem processes and disorders.

**Table 1:** The number of test questions answered by ChatGPT 3.5 and its performance, stratified by questions category (N=1840).

Question category	Test questions answered, n	Correct questions, n/N (%)
Male reproductive system	28	17/28 (60.1)
General principles and foundational science	29	16/29 (55.2)
Immune system	40	25/40 (62.5)
Skin and subcutaneous tissue	72	39/72 (54.2)
Renal and urinary systems	72	39/72 (54.2)
Biostats and epidemiology	87	45/87 (51.7)
Female reproductive system and breast	88	48/88 (54.5)
Musculoskeletal system	94	56/94 (58.5)
Endocrine system	103	58/103 (56.3)
Blood and lymphoreticular system	105	55/105 (52.4)
Pregnancy, childbirth, and puerperium	111	66/111 (59.5)
Behavioral health	115	73/115 (63.5)
Multisystem processes and disorders	122	73/122 (59.8)
Respiratory system	130	71/130 (54.6)
Social sciences	141	86/141 (61.0)
Gastrointestinal system	156	87/156 (55.8)
Cardiovascular system	161	89/161 (55.3)
Nervous system and special senses	186	104/186 (55.9)

**Table 2:** The number of test questions answered by ChatGPT 4 and its performance, stratified by questions category (N=229).

Question category	Test questions answered, n	Correct questions, n/N (%)
Endocrine system	1	1/1 (100)
Biostats and epidemiology	14	13/14 (92.3)
General principles and foundational science	17	14/17 (82.4)
Multisystem processes and disorders	17	15/17 (88.2)
Pregnancy, childbirth, and puerperium	19	15/19 (79.0)
Gastrointestinal system	21	18/21 (85.7)
Cardiovascular system	21	15/21 (71.4)
Nervous system and special senses	21	15/21 (71.4)
Blood and lymphoreticular system	23	20/23 (87.0)
Female reproductive system and breast	23	20/23 (87.0)
Behavioral health	24	21/24 (87.5)

### Test Question Length and ChatGPT Performance Scores

The mean character count was 1078 (SD 308). Test question length was significantly correlated with the performance of ChatGPT 3.5 (ρ=–0.069; *P*=.003) while not yielding significance for ChatGPT 4 (*P*=.87). For ChatGPT 3.5, the mean number of characters was 1062 (SD 310) for correct answers versus 1100 (SD 304) for falsely answered questions (*P*=.009). However, the mean character count was comparable for test questions answered by ChatGPT 4 (mean correct answers 1068, SD 274 vs mean false answers 1056, SD 233; *P*=.80).

### Test Question Difficulty and the Performance of ChatGPT

**Figure 1:**
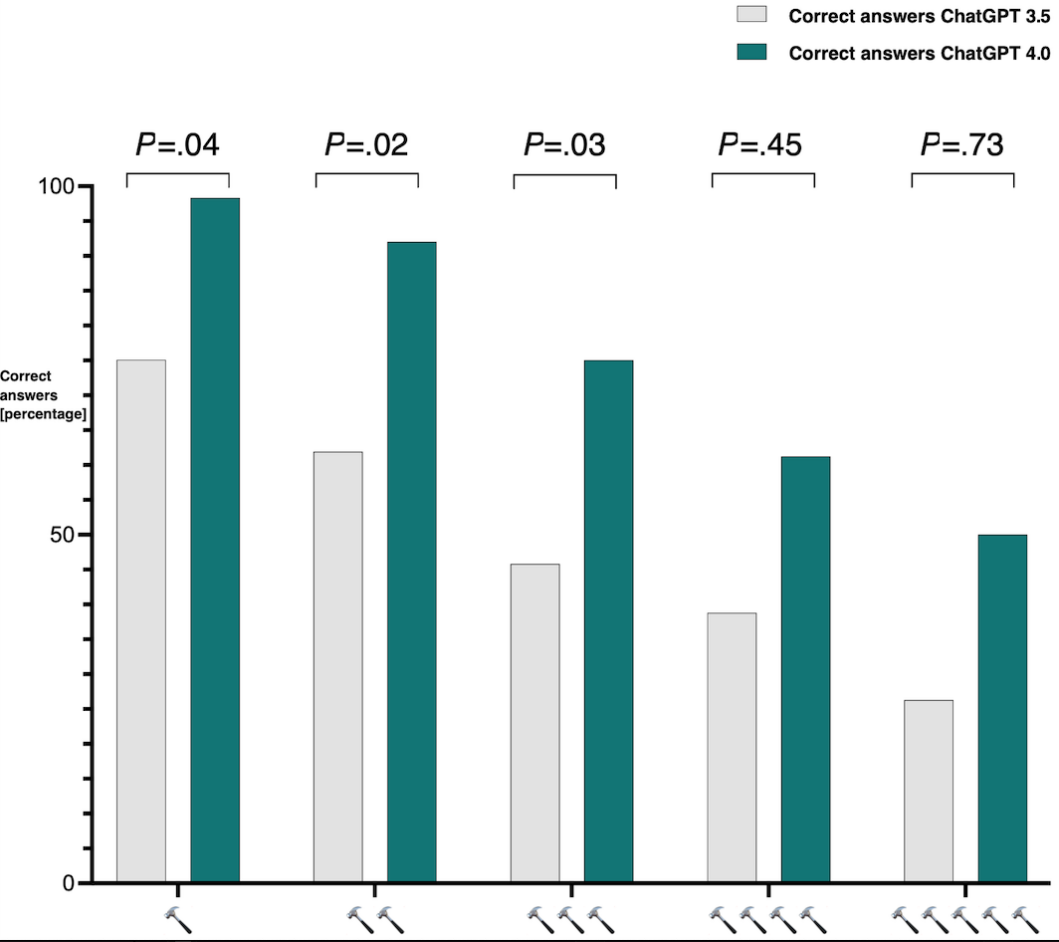
Question distribution and performance scores sorted by level of test question difficulty.

**Figure 2:**
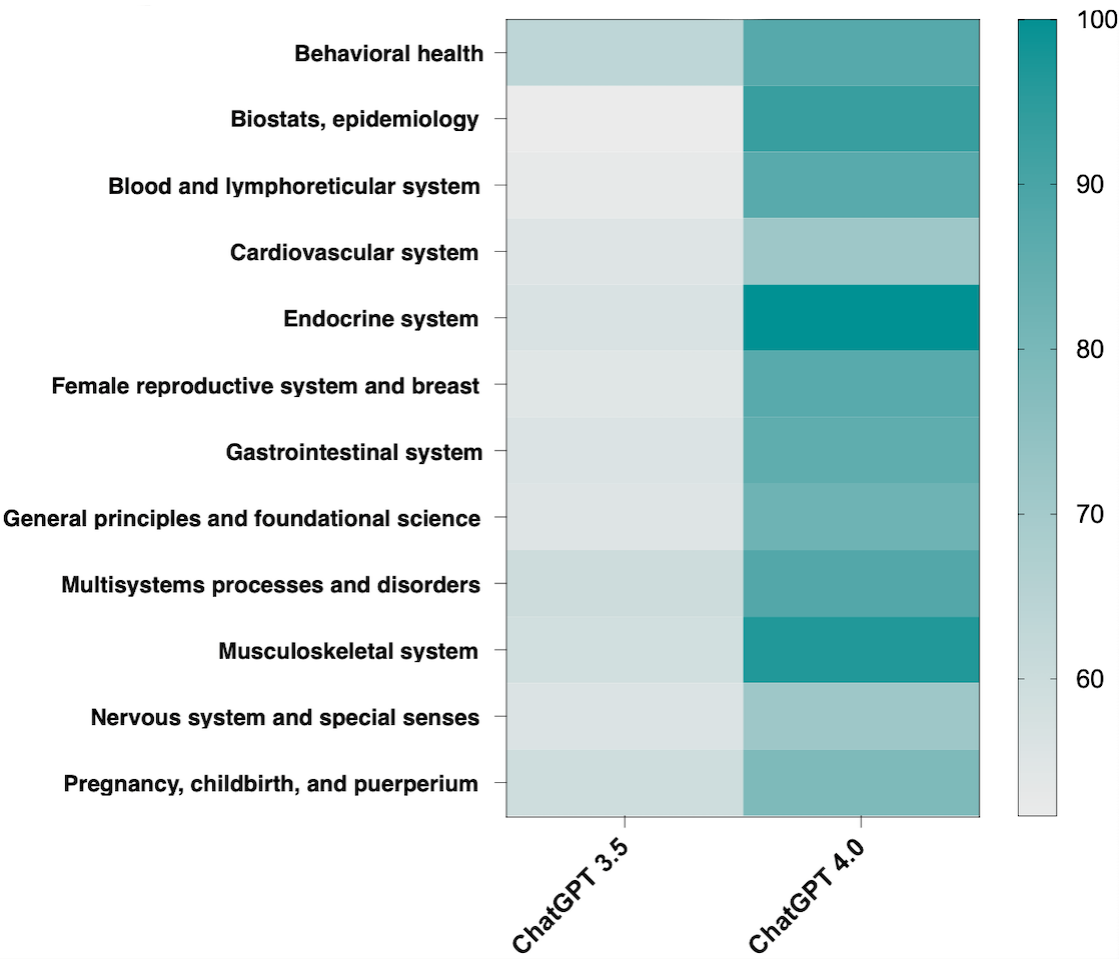
Percentage of correct responses from ChatGPT 3.5 versus ChatGPT 4.0, sorted by specialty.

Question distribution and performance scores sorted by level of test question difficulty are illustrated in Figure 1. Test question difficulty, defined by AMBOSS hammer categorization, and the performance of ChatGPT 3.5 were significantly correlated (ρ=–0.289; *P*<.001). This was reproducible in ChatGPT 4 (ρ=–0.344; *P*<.001). ChatGPT 4 statistically significantly outperformed ChatGPT 3.5 for each hammer category except for the 4- and 5-hammer test difficulty levels. For 1-, 2-, and 3-hammer questions, ChatGPT 4 had a statistically significant increase in accuracy compared to ChatGPT 3.5 (*P*=.04; *P*=.02; and *P*=.03; respectively). For the most difficult questions, ChatGPT 4 still had greater accuracy than ChatGPT 3.5; however, there was no statistical significance shown. The percentage of correct responses from ChatGPT 3.5 versus ChatGPT 4 sorted by specialty is illustrated in Figure 2. Relative to ChatGPT 3.5, ChatGPT 4 performed better on questions from every specialty category. The biggest differences in accuracy were in biostatistics, epidemiology, the endocrine system, and the musculoskeletal system.

### Buzzwords and the Performance of ChatGPT

**Figure 3:**
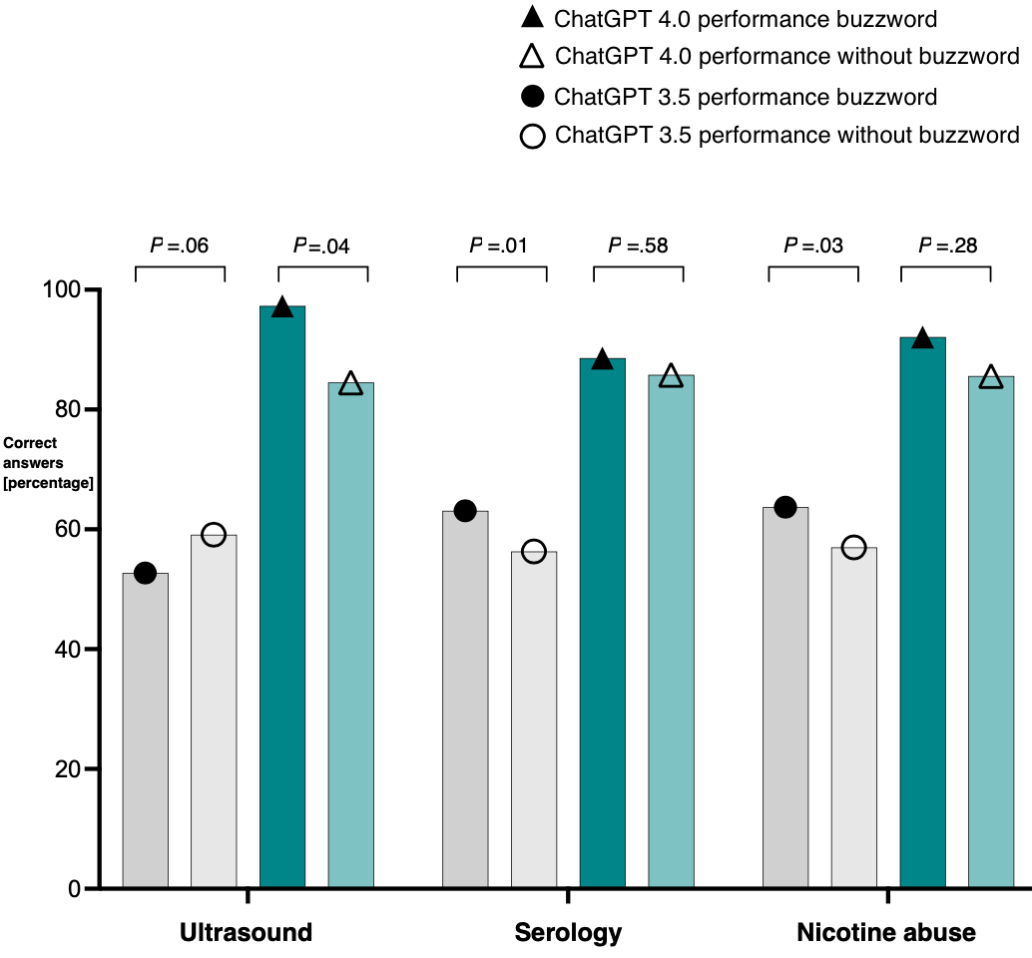
Performance scores of ChatGPT 3.5 versus ChatGPT 4.0, sorted by buzzword.

ChatGPT 4 performed significantly better on ultrasound-related questions (*P*=.04), while ChatGPT 3.5 answered significantly more questions correctly if they contained serology- or smoking-related information (*P*=.008 and *P*=.03, respectively). Performance scores of ChatGPT 3.5 versus ChatGPT 4 sorted by buzzwords are depicted in Figure 3. Overall, ChatGPT 4 outperformed ChatGPT 3.5, regardless of whether the question included buzzwords.

## Discussion

### Principal Findings

This investigation was designed to empirically evaluate and contrast the competencies of the 2 most contemporary iterations of the AI-powered large language model, ChatGPT, in relation to their performance in taking the USMLE Step 3. An aggregate of 1840 representative practice questions, derived from the AMBOSS question bank, were presented to ChatGPT version 3.5. The model delivered an overall accuracy rate of 56.9% (1047/1840). In juxtaposition, ChatGPT version 4 was assessed using a subset of 229 practice questions and achieved an overall accuracy rate of 84.7% (194/229). This difference in performance is both statistically and practically significant. Achieving a score of 84.7%, ChatGPT 4 falls within the top 10% of all test takers. In contrast, a score of 56.9% places ChatGPT 3.5 near the passing threshold. This significant difference provides empirical evidence of the substantial enhancements and refinements embedded within ChatGPT 4 and elucidates the leap in proficiency this iteration has attained, pushing the boundaries of AI capabilities in medical knowledge comprehension and application.

While ChatGPT 3.5 hovered around the approximate passing threshold of 60%, ChatGPT 4 not only passed the examination but merely excelled at it. According to the score interpretation guide provided by the National Board of Medical Examiners, an accuracy rate of 84.7% approximates placement within the 90th to 92nd percentile [14]. This signifies that ChatGPT 4 would be situated among the elite stratum, encompassing the top 10% of USMLE Step 3 candidates. The impressive escalation in performance exhibited by ChatGPT 4 makes the delineation of strengths and limitations difficult [15]. The model’s evolution seems to have attenuated discernible weaknesses, indicating a more well-rounded overall proficiency in the medical domain [12].

However, nothing is perfect. Although ChatGPT 4 accesses detailed, comprehensive, and up-to-date knowledge bases to optimize its response patterns, we could reveal minor performance weak points. We found that ChatGPT 4 was more prone to errors when answering test questions on cardiology (mean test accuracy: n=89, 71.4% vs n=15, 84.7% correct questions) and neurology (mean test accuracy: n=104, 71.4% vs n=15, 84.7% correct questions). Interestingly, these subjects often test the examinee’s transfer knowledge skills. Based on theoretical concepts (eg, Frank-Starling law and dermatome map), examinees are asked to filter the question stem for relevant patient data and adapt the underlying theory to the patient case. This novel insight into ChatGPT points toward persistent deficits in abstract thinking. Therefore, test question writers for the USMLE or other medical examinations may use this question style for other subjects to reduce the risk of AI cheating. Further, our analysis demonstrated that the performance of ChatGPT 4 significantly correlated (ρ=–0.344; *P*<.001) with the level of test question difficulty. This indicates that sophisticated USMLE questions still challenge and fool both human examinees and AI chatbots. Typically, the most difficult USMLE questions include distractors as well as irrelevant or additional information.; they also require high-level reasoning and interdisciplinary thinking. Our group previously showed that ChatGPT 3.5, similar to the human user peer group, struggled to answer 4- and 5-hammer questions [11]. Such pitfalls continue to perplex the next generation of AI-powered chatbots. Therefore, a thorough analysis of 4- or 5-hammer questions may help examiners refine their test questions and shield the USMLE against AI cheating.

Overall, the phenomenal improvement in the test-taking performance of ChatGPT 4 compared to ChatGPT 3.5 raises intriguing questions regarding future applications and implications of AI in medical education and diagnostics. AI has shown its prowess not only on the USMLE examinations in medical education but also on advanced examinations, such as the neurosurgical written boards [16]. This phenomenon ventures into other aspects of medicine as well, including research and clinical performance [17]. It is imperative that future research ventures into a deeper analysis of the performance of ChatGPT 4 by conducting thorough investigations that probe its strengths and limitations in a more granulated manner, potentially employing diversified medical question banks, simulating real-world scenarios, and engaging experts for analysis and evaluation to allow for the best possible medical education and ultimately patient health care [18].

### Limitations

This study needs to be interpreted in the light of the following limitations: first, due to the restricted use of ChatGPT (only 25 entries every 3 hours) we were not able to perform a direct comparison of ChatGPT 3.5 and ChatGPT 4 for all test questions included in this study, which might limit its validity. Furthermore, although we attempted to ensure that the questions provided for analysis were not freely available on the internet to minimize the risk of ChatGPT having already seen the exact question, students and researchers around the world may have input certain AMBOSS USMLE Step 3 Style Questions into ChatGPT. This adds a potential confounding factor of ChatGPT memorizing the correct answer from seeing the question beforehand. We used the 2 most recent versions of ChatGPT (ie, ChatGPT 3.5 and ChatGPT 4) to test and compare the performance of large language models on 1840 AMBOSS USMLE Step 3 questions. Thus, the findings of this study should be revalidated for upcoming ChatGPT versions. Future studies may involve additional chatbots, question banks, and image-based test questions. Further, the performance of ChatGPT on USMLE steps could be compared to other national medical licensing exams.

### Conclusions

This study is the first direct comparison of ChatGPT 4 and ChatGPT 3.5 based on 1840 AMBOSS USMLE Step 3 test questions. Our analysis showed that ChatGPT 4 outperformed its predecessor version across different specialties and difficulty levels, ultimately yielding accuracy levels of 84.7%. However, we could identify persisting weak points of ChatGPT 4, including abstract thinking and elaborated test questions. This line of research may serve as an evidence-based fundament to safeguard the USMLE steps and medical education against AI cheating while underscoring the potency of AI-driven chatbots.

## Abbreviations

AI: artificial intelligence
CK: clinical knowledge
CS: communication skills
MRI: magnetic resonance imaging
USMLE: United States Medical Licensing Examination
